# Supplementary material for: Multi‐dataset identification of innovative feature genes and molecular mechanisms in keratoconus
Source: J Cell Mol Med. 2024 Sep 19;28(18):e70079. doi: 10.1111/jcmm.70079 (PMC11412914; doi:10.1111/jcmm.70079)
Supplement: Supplementary file 2 — Material S2. [file JCMM-28-e70079-s002.zip › 7.GSEA analysis/1.ARL11/KEGG_BLADDER_CANCER.html]

Details for gene set KEGG\_BLADDER\_CANCER[GSEA]

|  || Dataset | ARL11.group.cls#h\_versus\_l |
| Phenotype | group.cls#h\_versus\_l |
| Upregulated in class | l |
| GeneSet | KEGG\_BLADDER\_CANCER |
| Enrichment Score (ES) | -0.3832422 |
| Normalized Enrichment Score (NES) | -1.0766194 |
| Nominal p-value | 0.4243028 |
| FDR q-value | 1.0 |
| FWER p-Value | 0.998 |
Table: GSEA Results Summary

  

Fig 1: Enrichment plot: KEGG\_BLADDER\_CANCER      
 Profile of the Running ES Score & Positions of GeneSet Members on the Rank Ordered List

  

| SYMBOL | TITLE | RANK IN GENE LIST | RANK METRIC SCORE | RUNNING ES | CORE ENRICHMENT || 1 | RPS6KA5 | na | 7067 | 0.110 | -0.1680 | No |
| 2 | CDKN2A | na | 8387 | 0.085 | -0.1785 | No |
| 3 | VEGFC | na | 8842 | 0.077 | -0.1666 | No |
| 4 | TP53 | na | 9447 | 0.068 | -0.1620 | No |
| 5 | FGFR3 | na | 9769 | 0.063 | -0.1508 | No |
| 6 | MYC | na | 10444 | 0.054 | -0.1526 | No |
| 7 | BRAF | na | 10628 | 0.052 | -0.1410 | No |
| 8 | KRAS | na | 10630 | 0.052 | -0.1242 | No |
| 9 | MDM2 | na | 10671 | 0.052 | -0.1086 | No |
| 10 | EGFR | na | 11115 | 0.046 | -0.1064 | No |
| 11 | PGF | na | 12205 | 0.034 | -0.1267 | No |
| 12 | EGF | na | 13590 | 0.022 | -0.1595 | No |
| 13 | VEGFB | na | 14052 | 0.018 | -0.1668 | No |
| 14 | TYMP | na | 15558 | 0.007 | -0.2079 | No |
| 15 | DAPK1 | na | 16033 | 0.004 | -0.2204 | No |
| 16 | ERBB2 | na | 16056 | 0.003 | -0.2200 | No |
| 17 | RB1 | na | 16201 | 0.002 | -0.2234 | No |
| 18 | E2F2 | na | 16509 | 0.000 | -0.2322 | No |
| 19 | CCND1 | na | 16546 | -0.000 | -0.2332 | No |
| 20 | CDH1 | na | 16747 | -0.002 | -0.2384 | No |
| 21 | MAP2K2 | na | 16997 | -0.003 | -0.2446 | No |
| 22 | E2F1 | na | 17721 | -0.008 | -0.2627 | No |
| 23 | HRAS | na | 18435 | -0.013 | -0.2790 | No |
| 24 | CDK4 | na | 18688 | -0.015 | -0.2814 | No |
| 25 | DAPK2 | na | 19907 | -0.023 | -0.3090 | No |
| 26 | DAPK3 | na | 21421 | -0.035 | -0.3415 | No |
| 27 | MAPK3 | na | 22614 | -0.046 | -0.3608 | No |
| 28 | RAF1 | na | 23336 | -0.054 | -0.3642 | Yes |
| 29 | RASSF1 | na | 23998 | -0.062 | -0.3631 | Yes |
| 30 | MAPK1 | na | 24626 | -0.071 | -0.3582 | Yes |
| 31 | E2F3 | na | 25055 | -0.077 | -0.3457 | Yes |
| 32 | THBS1 | na | 25706 | -0.086 | -0.3367 | Yes |
| 33 | ARAF | na | 26195 | -0.093 | -0.3206 | Yes |
| 34 | CDKN1A | na | 27494 | -0.119 | -0.3196 | Yes |
| 35 | NRAS | na | 27790 | -0.126 | -0.2875 | Yes |
| 36 | VEGFA | na | 28646 | -0.148 | -0.2643 | Yes |
| 37 | CXCL8 | na | 29280 | -0.165 | -0.2291 | Yes |
| 38 | MMP2 | na | 30057 | -0.190 | -0.1901 | Yes |
| 39 | MAP2K1 | na | 31070 | -0.223 | -0.1472 | Yes |
| 40 | MMP1 | na | 33608 | -0.370 | -0.1006 | Yes |
| 41 | MMP9 | na | 33942 | -0.411 | 0.0227 | Yes |
Table: GSEA details [plain text format]

  

Fig 2: KEGG\_BLADDER\_CANCER      
 Blue-Pink O' Gram in the Space of the Analyzed GeneSet

  

Fig 3: KEGG\_BLADDER\_CANCER: Random ES distribution      
 Gene set null distribution of ES for **KEGG\_BLADDER\_CANCER**
